# Data Visualization Support for Interdisciplinary Team Treatment Planning in Clinical Oncology: Scoping Review

**DOI:** 10.2196/69104

**Published:** 2025-12-09

**Authors:** Dominik Boehm, Cosima Strantz, Arsenij Ustjanzew, Iryna Manuilova, Alexander Scheiter, Thomas Pauli, Nicole Hechtel, Niklas Reimer, Jan Christoph, Hauke Busch, Thomas Ganslandt, Philipp Unberath

**Affiliations:** 1 Medical Center for Information and Communication Technology Universitätsklinikum Erlangen Friedrich-Alexander-Universität Erlangen-Nürnberg Erlangen Germany; 2 Chair of Medical Informatics Institute for Medical Informatics, Biometrics and Epidemiology Friedrich-Alexander-Universität Erlangen-Nürnberg Erlangen Germany; 3 Institute of Medical Biostatistics, Epidemiology and Informatics (IMBEI) University Medical Center of the Johannes Gutenberg-University Mainz Mainz Germany; 4 Junior Research Group (Bio-)Medical Data Science Faculty of Medicine Martin-Luther-University Halle-Wittenberg Halle Germany; 5 Institute of Pathology University of Regensburg Regensburg Germany; 6 Institute of Medical Bioinformatics and Systems Medicine Medical Center, Faculty of Medicine University of Freiburg Freiburg Germany; 7 Peter L. Reichertz Institute for Medical Informatics University of Braunschweig - Institute of Technology and Hannover Medical School Hannover Germany; 8 Institute for Systems Biology Lübeck Institute of Experimental Dermatology University of Luebeck Luebeck Germany; 9 SRH Fürth University of Applied Sciences Fürth Germany

**Keywords:** clinical oncology, tumor board, cancer conference, multidisciplinary, visualization, software, scoping review, tumor

## Abstract

**Background:**

Complex and expanding datasets in clinical oncology applications require flexible and interactive visualization of patient data to provide physicians and other medical professionals with maximum amount of information. In particular, interdisciplinary tumor conferences profit from customized tools to integrate, link, and visualize relevant data from all professions involved.

**Objective:**

Our objective was to identify and present currently available data visualization tools for tumor boards and related areas. We wanted to provide an overview of not only the digital tools currently used in tumor board settings but also of the data they include, their respective visualization solutions, and their integration into hospital processes.

**Methods:**

This scoping review was based on the scoping study framework by Arksey and O’Malley and attempted to answer the following research question: “What are the key features of data visualization solutions used in molecular and organ tumor boards, and how are these elements integrated and used within the clinical setting?” The following electronic databases were searched for articles: PubMed, Web of Science, and Scopus. Articles were deemed eligible if published in English in the last 10 years. Eligible articles were first deduplicated, followed by screening of titles and abstracts. Full-text screening was then conducted to decide on article selection. All included articles were analyzed using a data extraction template. The template included a variety of meta-information, as well as specific fields aiming to answer the research question.

**Results:**

The review process started with 2049 articles, of which 1014 (49.49%) were included in the title and abstract screening. A total of 5.47% (112/2049) of the publications were eligible for full-text screening, leading to 2.93% (60/2049) of the publications being eligible for final inclusion. They covered 49 distinct visualization tools and applications. We discovered a variety of innovative visualization solutions, most often driven by the complexity of omics data, represented in 96% (47/49) of the tools. Tables remained the most used tool for the visualization of data types described in the articles. Approximately one-third of the identified tools (16/49, 33%) were systematically evaluated in some form. For most discovered tools (37/49, 76%), there was no documentation of implementation into the clinical routine. A significant number of applications (21/49, 43%) were available through open-source access.

**Conclusions:**

There is a wide range of projects providing visualization solutions for tumor boards and clinical oncology applications. Among the few tools that have made their way into clinical routine settings, there are both commercial and academic solutions. While tables for a variety of data types remain the dominant visualization strategy, the complexity of omics data appears to be the driving force behind many visualization innovations in the domain of tumor boards.

**International Registered Report Identifier (IRRID):**

RR2-10.2196/53627

## Introduction

### Background

Cancer care, especially in cases of advanced disease, requires coordinated multidisciplinary approaches. Tumor boards are oncological case conferences that allow clinicians from multiple specialties to examine patient data together and determine the optimal path of treatment going forward. Interventions by these multidisciplinary teams, particularly through tumor boards or multidisciplinary cancer conferences (MCCs), have demonstrated substantial improvements in cancer care quality [1-3]. MCCs historically started as weekly meetings of oncologists, surgeons, radiologists, and professionals from other disciplines discussing complex oncological cases to determine the best treatment going forward. With increased research and the resulting increased specificity, organ tumor boards emerged. These focus on cancers within particular organ systems, allowing for more personalized treatment from clinicians who are experts in current therapies and clinical trials for those cancers [4]. Molecular tumor boards (MTBs) are the latest development in personalized cancer medicine. They allow clinicians to analyze the cancer’s molecular profile and explore additional treatment options based on observed genomic alterations after collecting and sequencing a patient’s cancer samples [5]. The challenge of visualizing complex, multimodal data that are continually expanding is especially notable in these multidisciplinary environments [5]. These data encompass a wide range, from demographic details and laboratory findings to tumor imaging, therapeutic timelines, and genomic information, offering extensive possibilities for combined visualization and data aggregation. The critical need for digital tools and tailored visualization solutions becomes clear given the stringent time limitations that are common in clinical oncology. Treatment decision periods are often constrained to 10 to 20 minutes per patient [6], underscoring the necessity for well-aggregated and annotated data that allow health care professionals to incorporate all pertinent details into their decision-making processes. Beyond the time constraints, the increasing complexity of patient data makes it difficult to fully understand a patient’s health status without effective visualization support, particularly when dealing with multiple data points along the patient’s journey [7,8]. Although tools for visualizing such multimodal data do exist in these settings [9-11], there is currently no comprehensive overview of the actively used and established visualization tools, as well as their distinct features on a global level.

Previous research has shown that digital applications may support a variety of the processes in MCCs, ranging from standardized access to databases and digital support for literature and trial search to enhance case preparation to the structured documentation of MCC results [12]. The emergence and evolution of MTBs further highlight the need for advanced visualization tools. They require the integration of large amounts of genomic data with conventional clinical information and demand robust visualization tools to assist participating medical professionals in making informed treatment decisions efficiently [13,14]. With the shift toward virtual MTBs and an increase in outpatient referrals, the demographic of tumor board participants has broadened to include a more diverse group, encompassing various specialties and sometimes spanning different languages. This shift requires visualizations that are both intuitive and comprehensive, accommodating participants’ varied levels of familiarity with each patient’s history [15]. To address these needs, specialized software platforms such as cBioPortal [16-18], The Cancer Core Europe Molecular Tumor Board Portal [19], and Alteration Annotations for Molecular Tumor Boards (AMBAR) [20] have been developed, providing detailed visual representations of genomic data in cancer samples.

In addition to these platforms, resources such as OncoKB [21] and Clinical Interpretation of Variants in Cancer [22] have been established. These knowledge bases deliver structured and aggregated information concerning targeted therapies, which is crucial for maintaining consistency in therapeutic recommendations across tumor boards, especially for patients with rare cancers or unique mutation patterns [23]. Moreover, research indicates that variability in the tools and methodologies used can lead to inconsistencies in therapy recommendations [24], further underlining the need for standardization.

Due to the heterogeneous nature of data formats and application interfaces, integrating data from various sources to enable comprehensive visualization is a challenging task, often requiring custom extract, transform, and load workflows to homogenize the data and convert them into a shared format [25]. Depending on the data concerned, this is further complicated by data privacy regulations [26]. Initiatives such as the Medical Informatics Initiative [27], the Bavarian Center for Cancer Research [28], and the German Network for Personalized Medicine [29] spearhead efforts in Germany to standardize datasets and processes to better facilitate decision-making within the health care sector. These initiatives are also investing resources to improve existing tools such as cBioPortal, enhancing their capabilities to document and visualize therapeutic decisions during MTBs [30,31].

### Objectives

Given the described circumstances and the increasing complexity and volume of data in clinical oncology, there is a rising need for advanced visualization tools that effectively leverage these data for decision-making in tumor board settings. Integrating such software solutions into existing clinical workflows is challenging yet essential as it ensures that data from various sources are readily accessible for effective use in tumor boards. This scoping review aimed to explore and describe the available visualization support for MCCs, outline the key visualization strategies used, and examine their accessibility as well as their integration into clinical processes. The goal was to provide a comprehensive overview of the current landscape and future directions of digital support tools in tumor board settings.

## Methods

### Design

This scoping review was conducted using the scoping study framework by Arksey and O’Malley [32] as a methodological blueprint. Arksey and O’Malley [32] describe a five-step model for the design of scoping studies: (1) identification of the research questions; (2) identification of relevant studies; (3) study selection; (4) data extraction and charting; and (5) collating, summarizing, and reporting the results. A scoping review protocol was created and published in preparation for the review describing the study using these steps [33]. The following sections will highlight any deviations from the described templates and processes.

### Core Concepts and Keywords

In an initial manual literature search, 19 key papers were identified and used to establish core concepts and potential keywords for the development of a search strategy (Table 1). We were able to determine that viable literature needed to encompass 3 concepts: first, our target domain of choice, tumor boards and multidisciplinary conferences; second, software or some other form of digital support; and, finally, some kind of support or visualization system.

**Table 1:** Concepts and corresponding keywords.

Concept	Keywords
Target domain	Tumor board, tumor conference, MTB, mutation database, and cancer genomics
Software	Virtual, digital, software, tool, platform, and portal
Mode of support	Visualization, interactive, preparation, usability, clinical decision support system, personalized medicine, and precision medicine

### Search Strategy

Our search strategy included the established electronic databases PubMed, Scopus, and Web of Science. Individualized queries were designed; tested; and, finally, validated by a research librarian. The detailed query development is described in the scoping review protocol [33]. In addition, the individual queries can be found in Multimedia Appendix 1.

### Eligibility and Screening

Before data extraction and analysis, all initially included articles were subjected to 2 rounds of screening. First, title and abstract screening was conducted by at least 2 blinded reviewers (DB, CS, AU, IM, AS, TP, NH, NR, and PU). In case of conflicting decisions, an additional reviewer (JC) was included for a majority decision [34]. Second, all articles were subjected to a full-text screening, examining the entire publication’s contents for relevance to the review. Only articles published in English in the last 10 years were included. As an initial search showed a large variety of target literature types, we decided not to use any further meta–inclusion or exclusion criteria to include all potentially relevant articles. The objective was to include articles in the review that provided information on the visualization solutions used in molecular and organ tumor boards. More specifically, they should be able to (partially) answer the overarching research question: What are the key features of data visualization solutions used in molecular and organ tumor boards and how are these elements integrated and used within the clinical setting?

Starting from this overarching research question, specific questions that we wanted to answer while extracting data from relevant literature were developed. These were used at a later step to design the data extraction template:

1. What data visualization solutions are being used?
2. What kind of data are being visualized?
3. How do these tools visualize the available data?
4. How are these elements integrated and used within the clinical setting?
5. How accessible are the solutions?
6. Have the proposed or implemented solutions been evaluated?

The inclusion and exclusion criteria, summarized in Textbox 1, were defined in extensive discussions among all reviewers and pretested before each screening phase. All screening phases were conducted using Rayyan (Qatar Computing Research Institute) [35] to structure and document inclusion decisions. Data extraction was then performed by a single reviewer per article.

**Inclusion and exclusion criteria for the scoping review.**

**Inclusion criteria**

- Study types: any type of published peer-reviewed original research using qualitative, quantitative, or mixed methods
- Period: any article published between 2013 and 2023 (inclusive)
- Language: English
- Concept: any study that included applications with visualization features in the domain of molecular and organ tumor boards or in general

**Exclusion criteria**

- Period: thematic articles published before 2013
- Language: studies published in languages other than English

### Data Extraction, Collation, and Synthesis

All included articles were subject to the data extraction process using an extraction template that included meta-information as well as information on the found data types and their respective visualization solutions. Before the data extraction process, participating researchers discussed the template to ensure consistent extraction and comparable categorization and to limit the amount of processing needed. Afterward, the data extraction–identified tools were subjected to an additional manual literature search to close potential data gaps.

Following data extraction and charting, the results were analyzed in a 2-step process following the framework by Arksey and O’Malley [32]. First, the findings were analyzed comparing the extent, nature, and distribution of the identified articles. We also prepared a thematic overview on visualization solutions for tumor boards and clinical oncology. The visualized data per tool, as well as the respective visualization strategies used and remaining elements of the data extraction template, were charted, and appropriate graphics were created.

### Ethical Considerations

As our review did not involve human participants, no ethics approval was required for this study.

## Results

### General Results

**Figure 1:**
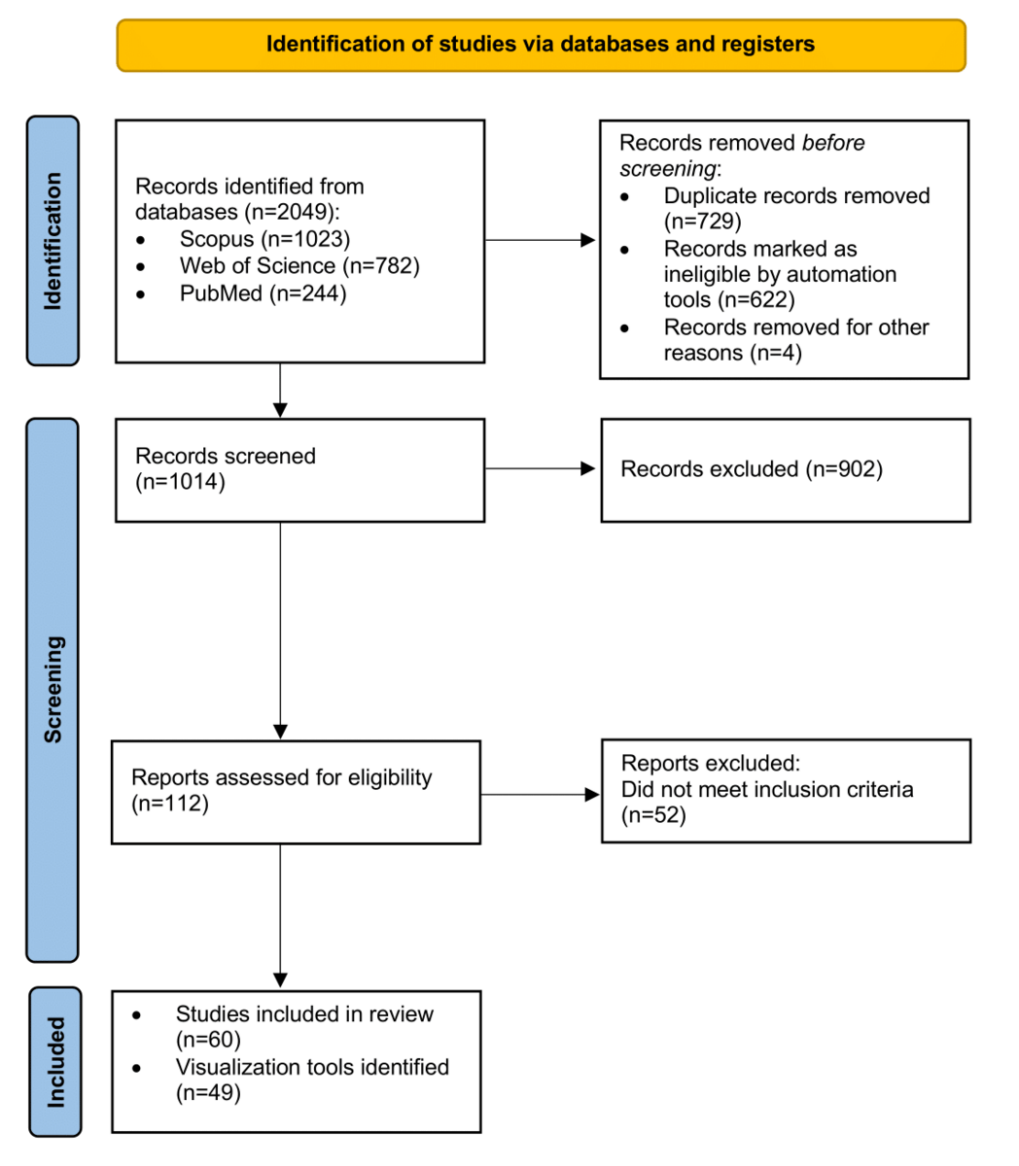
PRISMA (Preferred Reporting Items for Systematic Reviews and Meta-Analyses) flowchart [36]. Removal of duplicate records and records removed through automation tools before screening are overlapping categories.

The review process started with 2049 articles, of which 1014 (49.49%) were included in the title and abstract screening. A total of 5.47% (112/2049) of the publications were deemed eligible for full-text screening, resulting in 2.93% (60/2049) of the publications being suitable for final inclusion. The process was documented in a PRISMA (Preferred Reporting Items for Systematic Reviews and Meta-Analyses) flowchart (Figure 1) [36]. The included articles covered 49 distinct visualization tools and applications. The distribution of relevant publications over the last 10 years was relatively consistent, with a slight decline before 2016 and an overall upward trend (Multimedia Appendix 2). The articles were almost exclusively original research articles (58/60, 97%), followed by reviews (2/60, 3%).

### Overview: Data Types and Visualization Strategies

Most of the identified visualization tools (47/49, 96%) were capable of processing omics data, including primarily a variety of genomics data but also proteomics and transcriptomics data. The second most prevalent type of data was clinical data (31/49, 63%), followed by annotations with external data sources (19/49, 39%).

**Figure 2:**
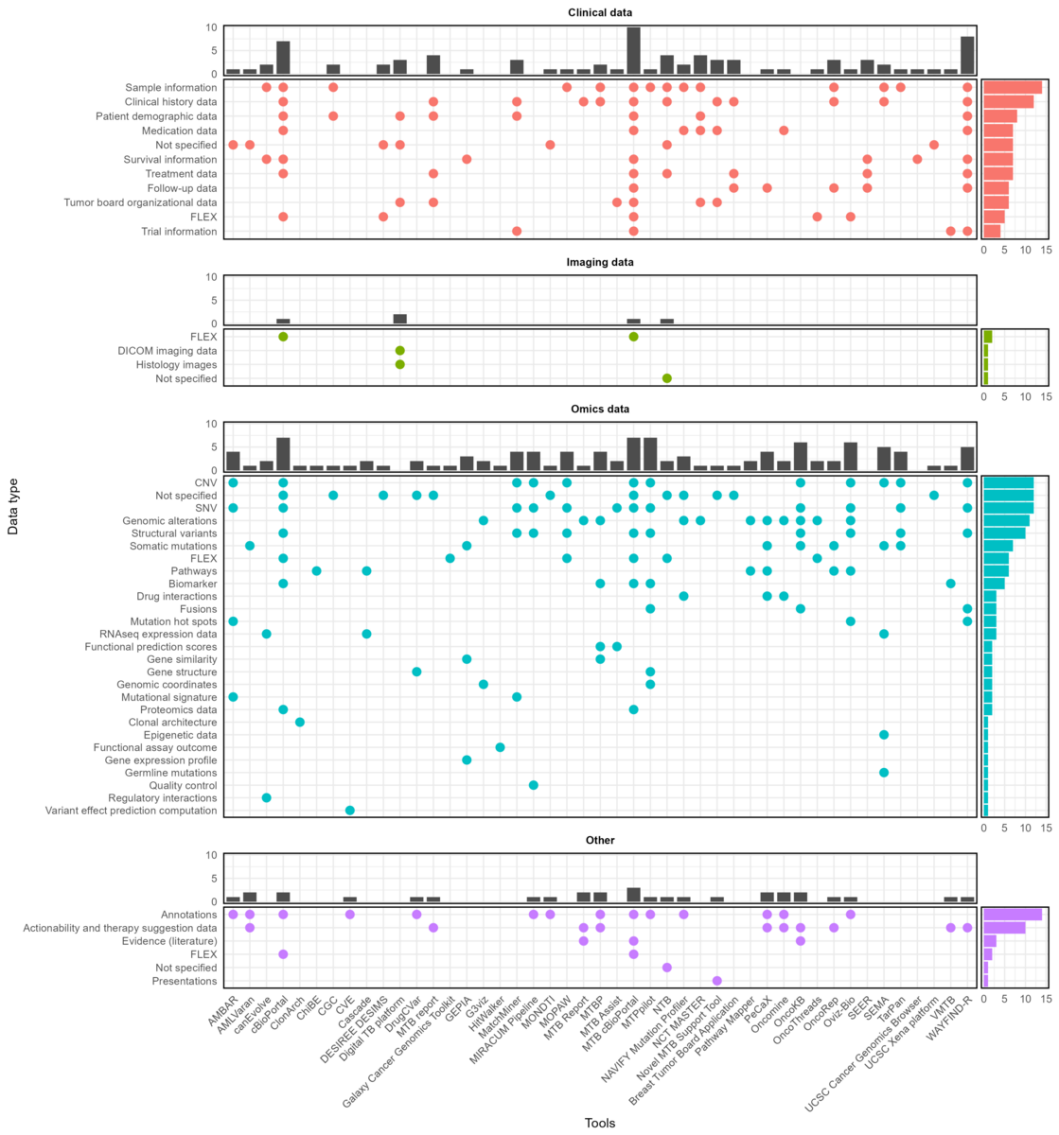
Data type overview. Filled fields represent the existence of visualization for the data type. FLEX rows represent generalized visualization solutions for the category that can be primarily used through user-defined data. CNV: Copy Number Variation; DICOM: Digital Imaging and Communications in Medicine; SNV: single nucleotide variant. Tool acronyms: AMBAR (Alteration Annotations For Molecular Tumor Boards), AMLVaran (AML Variant Analyzer), ChiBE (Chisio BioPAX Editor), CGC (Cancer Genomics Cloud), CVE (Cancer Variant Explorer), MTB report (From somatic variants towards precision oncology: Evidence-driven reporting of treatment options in molecular tumor boards), GEPIA (Gene Expression Profiling Interactive Analysis), MOPAW (Multi-omics Pathways Workflow), MTBP (Molecular Tumor Board Portal), MTPpilot (Molecular Tumor Profiling pilot), NTB (NAVIFY Tumor Board), Novel MTB Support Tool (User-Driven Development of a Novel Molecular Tumor Board Support Tool), Breast Tumor Board (A Digital Solution for an Advanced Breast Tumor Board: Pilot Application Cocreation and Implementation Study), PeCaX (personalized cancer network explorer), SEER (Surveillance, Epidemiology and End Results), TarPan Viewer (Targeted Panel Viewer), VMTB (virtual molecular tumor board).

**Figure 3:**
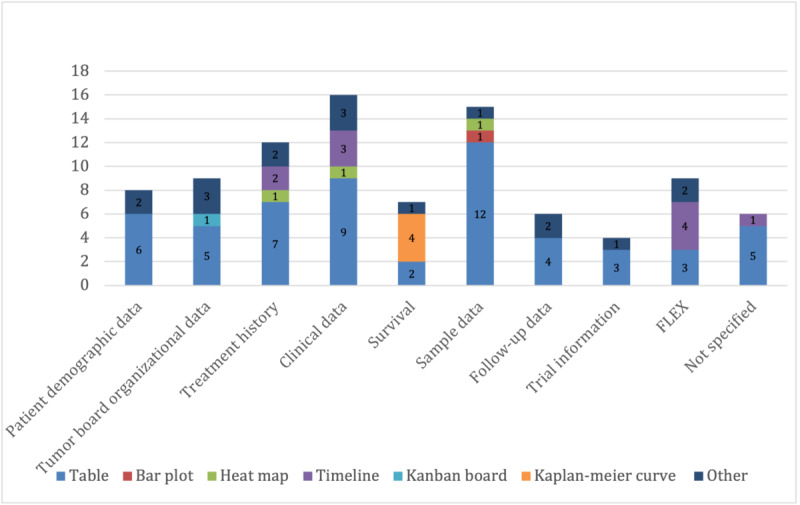
Distribution of the data types and visualization strategies for patient-related data.

**Figure 4:**
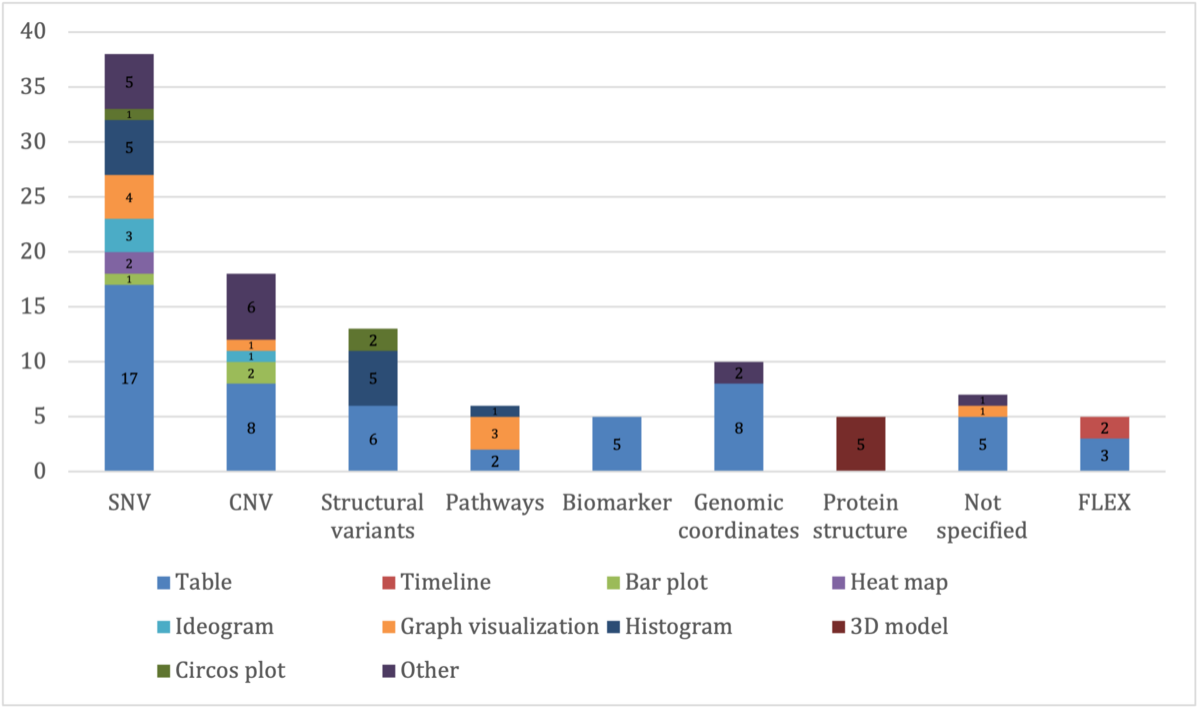
Distribution of data types and visualization strategies for omics data. CNV: copy number variation; SNV: single nucleotide variant.

**Figure 5:**
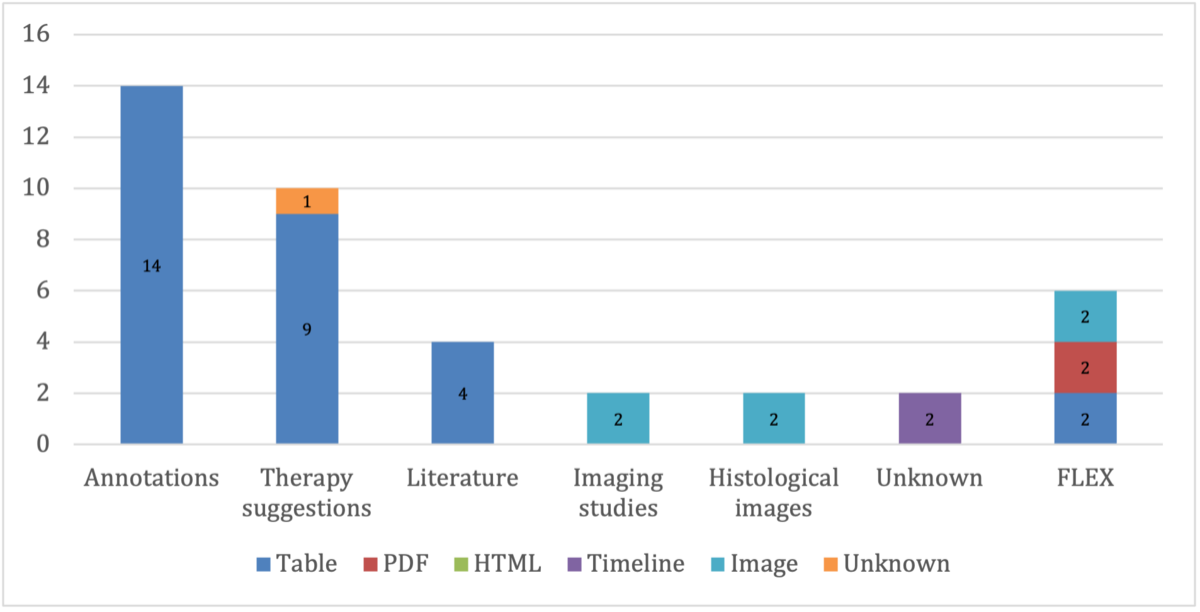
Distribution of the data types and visualization strategies for miscellaneous data.

Of particular interest was the mapping between data types and visualization strategies (Figures 2-5) as it revealed the driving forces behind new developments, as well as implicit user requirements. While (interactive) tables remained the most frequently used form of visualization for MTB data, omics data and multidisciplinary applications provided incentives for new visualization solutions, as well as for the joint visualization of multiple data types. The following sections present the most common or noteworthy data types and visualization techniques.

### Patient-Related Data

The most commonly available and visualized patient-related data were clinical (16/49, 33%) and sample (15/49, 31%) data (Figure 3), with diagnosis data being the most prevalent. These were mostly presented in the form of tables summarizing existing clinical information. Treatment history was also readily available (12/49, 24%) and included information on medications and treatment metadata. Another notable visualization technique was timelines, often representing treatment history. The remaining visualization types—for example, heat maps—primarily visualized the co-occurrence of clinical data with other data types, especially omics data (Figure 4). A small but significant number of tools (6/49, 12%) also included organizational data of tumor boards, covering session information, participants, and resulting decisions. These tools were mostly designed with a focus on software support for tumor board meetings. A significant number of tools (9/49, 18%) also enabled users to supply flexible custom data for visualization. This was primarily available in applications that offered timeline visualization, such as OncoThreads.

### Omics Data

Omics data, but especially genomic data, were the most commonly available data type, with single-nucleotide variants being visualized in approximately two-thirds of the applications discussed (38/49, 78%), followed by copy number variations (18/49, 37%) and structural variants (13/49, 27%). In addition, there was a considerable number of tools (16/49, 33%), such as PathwayMapper [37], that offered innovative visualization solutions for 1 specific data type.

The data were visualized using a variety of techniques (Figure 4), with tables still being the most prevalent technique. However, in comparison to clinical data, signaling pathways were consistently presented using graphical visualization techniques. Protein structures were usually represented using 3D models. Some applications integrated omics data into their timeline features depending on how much chronological data were available. In addition to patient-related clinical data and omics data, a range of other data types were visualized (Figure 5).

These ranged from imaging studies to annotations from external data sources, with annotations (14/49, 29%) and therapy suggestions (10/49, 20%) being the most common. As expected, imaging studies and histological images were generally simply displayed without additional visualization functions.

### Integration of Visualization Solutions Into the Clinical Setting

In general, there was a lack of integration of stand-alone applications into digital hospital systems. However, some tools used semistandardized data formats; only standards such as the Health Level Seven–Fast Healthcare Interoperability Resources were used.

Nevertheless, a lot of emphasis was placed on integration into clinical and research processes. Even though many tools primarily focused on research applications, those that aimed to play an active role in the presentation or documentation of tumor board content, such as MTPpilot [10], MTB-cBioPortal [30], or the Digital TB Platform [37], proposed solutions to some of the challenges faced by multidisciplinary conferences. These applications also tended to use structured evaluations to ensure the quality and process enhancements of the developed tools.

### Accessibility

Most applications (21/49, 43%) were available with open-source access. Often overlapping with this group, several tools (9/49, 18%) were also accessible in a container format. This almost exclusively refers to the availability of Docker containers. Web applications (12/49, 24%) were also quite common. For a similar number of tools (12/49, 24%), there was no information available, or it was not accessible. There were also a small number of commercial tools (3/49, 6%), namely, the navify Tumor Board [38], the navify Mutation Profiler [39], and WAYFIND-R [40], that were part of academic publications.

### Existing Use in Hospitals

A smaller proportion of the described visualization tools (8/49, 16%) were already established in clinical routine (eg, the MTB-cBioPortal and Molecular Tumor Board Portal [41]). Although there were additional solutions (4/49, 8%), including canEvolve [42] and TarPan [43], that were used exclusively in hospital research, there was no documentation of actual clinical implementation for most (37/49, 76%).

### Systematic Evaluation

Approximately one-third of the identified tools (16/49, 33%) were systematically evaluated in some form. However, the quality and quantity of the evaluations were very heterogeneous. While the systematic evaluation was the focus of some articles covering different tumor boards and types of cancer, other publications focused on different topics. However, all articles included in the group that carried out systematic evaluations at least conducted structured interviews with users.

While the heterogenous nature of the evaluations makes direct comparisons difficult, several interesting individual observations were made. The tool for tumor board support described by Nobori et al [37], for example, was able to cut the time investment for case preparation almost in half. They also observed that more than 90% of the surveyed clinicians preferred the digital tumor board application over the established manual process. Hammer et al [38] described varying results across several evaluated tumor boards but were still able to show a significant decrease in case discussion time. Their multistage evaluation was also able to indicate that the benefits of implementation increased over time. Even though the distinct methods and results varied, the outcomes followed the same trend: visualization support for tumor board preparation and execution reduced the resources required while enhancing the process for participating clinicians.

### Geographical Origin

We also examined the geographical distribution of the original articles based on the first authors’ primary affiliation (Multimedia Appendix 3). The highest number of articles originated in the United States (25/60, 42%), with Germany in second place (16/60, 27%). This was followed by China (4/60, 7%), Canada (3/60, 5%), the United Kingdom (3/60, 5%), and France (3/60, 5%). The remaining articles (6/60, 10%) originated mostly in European countries. No significant trends in the data types used could be identified. A general trend favoring omics data was consistent across all origins, as has been described in a previous Results subsection. However, European—and particularly German—articles referenced significantly more applications for organizational support of tumor boards, for example, MTB-cBioPortal [30] and AMBAR [20]. Meanwhile, articles from the United States more often described tools for unique visualization approaches and exploration of datasets of varying data types.

### Tool Categories

**Figure 6:**
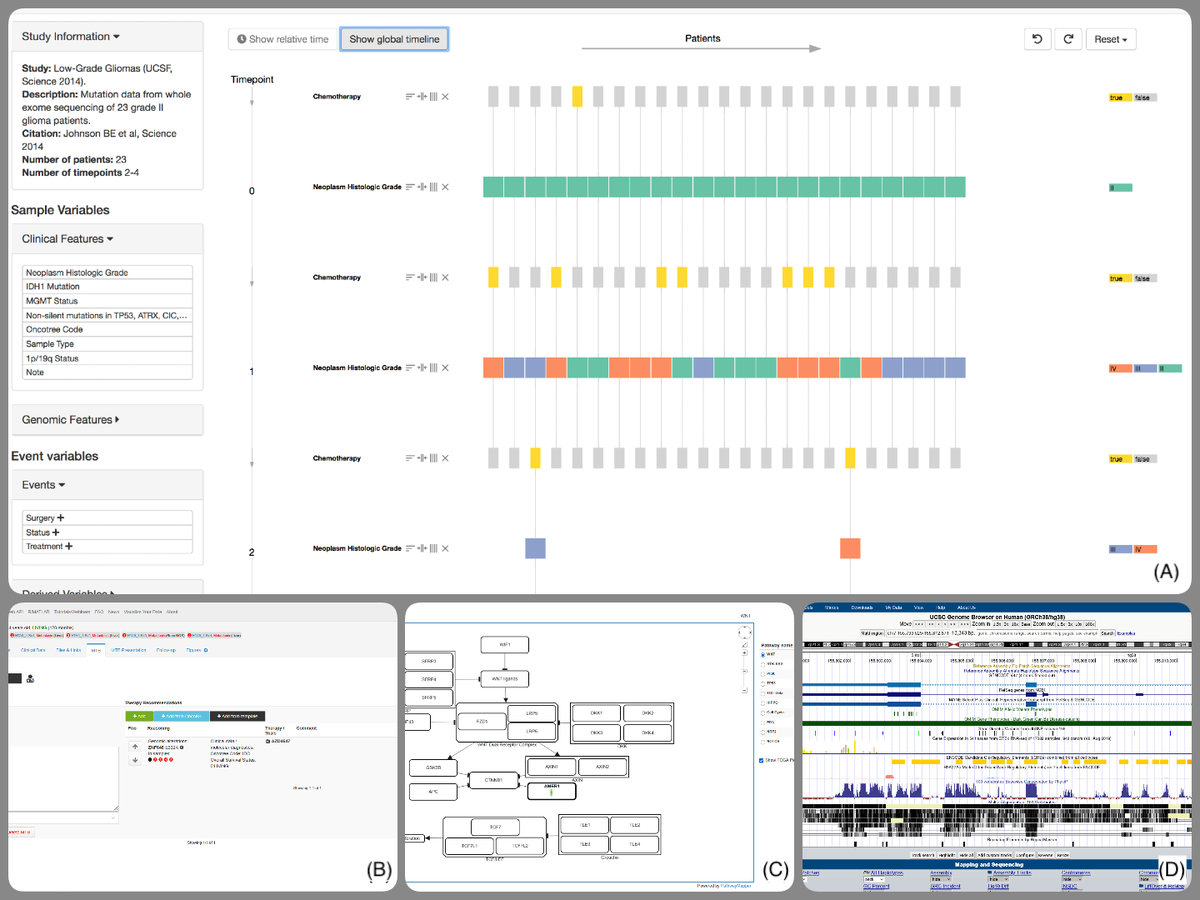
Tool category examples: (A) Joint data visualization in oncothreads; (B) Organizational visualization through the Molecular Tumor Board-cBioPortal recommendation documentation; (C) Multi-tool integration in the cBioPortal pathway view; (D) Specialized visualization using the University of California Santa Cruz Genome Browser.

While there was often significant overlap between the capabilities of the identified visualization tools, we were able to identify 4 general visualization tool groups (Figure 6). The first was joint data visualization, displaying a combination or overview of available data, often across multiple patients and sometimes including a temporal component. This group encompassed tools such as AMBAR [20], WAYFIND-R [40], and OncoThreads [44], and the tools tended to be used for either cohort analysis or active support during case preparation and presentation for tumor boards. The second group was organizational support applications that offered documentation features or focused on tumor board metadata, such as process steps and participants. Examples of this are the MTB-cBioPortal extensions [30] or the Digital TB Platform [37]. The third group consisted of applications such as cBioPortal [16] that incorporate other specialized tools to offer a more comprehensive toolkit for the visualization of joint data. The last group were specialized visualization tools. These offered often unique visualization features for specific data types and may be incorporated into tools from the third group. PathwayMapper [45], G3viz [46], and the University of California, Santa Cruz, Genome Browser [47] are examples from this group.

## Discussion

### Principal Findings

The results of this scoping review show the key aspects of data visualization solutions in the realm of tumor boards and clinical oncology. We were able to extract 60 relevant publications from the body of literature, in which 49 distinct tools and applications were discussed. Over the past 10 years, there has been substantial and consistent work on visualization issues, especially driven by growing datasets and novel data types in omics applications.

This is also reflected in the distribution of visualization tools by data type (Figures 3-5). There is a significant gap between the complexity of visualization for omics data and that of all other data types. In this paper, we can only speculate as to why this is the case as there are numerous and diverse drivers for innovation in the field of visualization [48]. We assume that highly complex biomedical information—such as omics data—is understandable to medical professionals in their field even without visualization aids. However, in contrast, there is a need for additional visualization solutions to increase the ability to transfer information quickly so that a multidisciplinary team can draw reliable conclusions based on these data, especially in a limited time frame.

Another interesting observation was the lack of innovation with respect to clinical data. While visualization techniques for other data types were rather widely used, clinical data were almost exclusively communicated in the form of tables. This may be for several reasons. First, this is perhaps the optimal form of visualization for most applications. However, it could also reflect a resistance to change or a lack of awareness of alternative, more dynamic visualization methods that could enhance data interpretation and decision-making or the interactivity of table components, for example, AMLVaran [49]. However, interactivity is not limited to table components offering additional information on hovering or using basic search functions. Even complex visualization techniques such as phylogenetic trees used by clonarchs [50] to visualize the phylogenetic similarity between clones deploy interactivity to enable the user to select which data to visualize and focus on specific relationships or data features.

One of the key accessibility issues faced by many tools is the transformation from research projects into routine applications. This requires constant maintenance and, therefore, continuous funding. Promising projects such as MAGI [51] were published and, at some point, stopped receiving updates. This unfortunate circumstance presents a resource problem as often applications are developed as prototypes without the extensive design and planning that are required to create maintainable software. While this is unavoidable to some extent, these issues can be mitigated by creating reusable software and making it accessible via an application programming interface or as a module that may be integrated into or with other projects. The feasibility of this approach is evident when looking at applications such as cBioPortal that incorporate and use several tools that supply visualization features for specific tasks.

An additional hurdle is the lack of inclusion of visualization tools into routine clinical processes. This may be due to several factors; however, we assume interoperability and data integration to be the main obstacles. Many of the tools we found were based on varying and proprietary data types. Transforming available data from heterogeneous electronic health record systems for every application is a resource-intensive task that may not justify the cost. Ensuring availability of the necessary data in the required time frame for tumor board sessions is additionally difficult. Bundling unique tools into toolkits such as cBioPortal or the University of California, Santa Cruz, Genome Browser with joint data interfaces may increase the benefit of extract, transform, and load development and lower the barriers to process integration. In addition, the development and adoption of national and international standards for health care data, such as Fast Healthcare Interoperability Resources, show great promise in improving the interoperability between hospital systems and new visualization tools.

Of special interest to us were potential visualization solutions for patient-reported outcome measures and questionnaires in general. However, unfortunately, no such visualization strategies were described. We assume that this is primarily due to a general lack of data as follow-up information is generally difficult to obtain and digital representation of it outside of research settings is currently rare.

### Limitations

The described review process and its findings have a few key limitations. First, there was a dependency on academic publications covering the visualization solutions that we aimed to include. This means that commercial solutions that might offer important insights may have been overlooked if they were not mentioned in a research article. In general, commercial solutions are a lot more restrictive regarding the amount of publicly available information. In addition, the decision to only include articles in English potentially omitted several articles that might have been relevant to the research questions. We identified fewer than 100 non–English-language publications, which we chose to exclude due to the additional effort required for translation and interpretation. Given that our search queries yielded a limited number of non–English-language articles, we considered this exclusion acceptable. Second, the quality of the information contained in the source publications varied wildly. Visualization features were often described only briefly or as a side note. While it is not feasible to describe every reaction to a mouse click, the discussion of visualization and user interface capabilities was often replaced by a single screenshot. This is unfortunate as these components play a key role in the usability of innovative applications and are a necessity when it comes to user acceptance. Third, due to the often experimental nature of (molecular) tumor boards, some tools were included in this review that lay on the boundary between biomedical research and clinical application. Nevertheless, their potential ability to communicate complex information efficiently, especially in the field of omics, was deemed important by the research team. Given that tumor boards are multidisciplinary conferences that require all participants to comprehend the information provided, strong visualization supporting the explanation of—for example—relevant pathways and other disease mechanics is essential. Finally, visualization and user interface components are, in turn, highly flexible and, therefore, heterogeneous. Due to this, comparing different tools requires a certain degree of generalization, which may affect the grade of detail at the individual tool level. That said, we consider this a necessary step to offer a relevant overview of the current state of visualization solutions for tumor boards and clinical oncology.

### Comparison With Prior Work

A narrative review by Abudiyab and Alanazi [52] explored the role of data visualization techniques in health care, focusing on their application, benefits, and future directions. Data visualization encompasses various forms, such as graphs, charts, and diagrams, aiding health care providers in understanding trends and making informed decisions. The review used a descriptive analysis methodology, sourcing articles from databases such as PubMed and Google Scholar. Visualization techniques are crucial in modern health care due to the increasing volume and complexity of data. As defined by Gartner [53], interactive visualization can be understood as the manipulation of graphical information through color, brightness, movement, and geometry to elevate the meaning of the data presented. Historical context highlights Florence Nightingale’s pioneering use of visualization for health care data in the 19th century. The aforementioned review underscored several benefits, including improved patient care and disease trend recognition, simplified data presentation, accelerated performance, and error detection.

Scheer et al [54] conducted a scoping review of visualization solutions with a focus on time-oriented data. Information visualization and visual analytics simplify complex time-oriented patient data, enabling focus on underlying patterns. This review aimed to identify visualization techniques for time-oriented health care data, facilitating patient comparison. Using the PRISMA-ScR (Preferred Reporting Items for Systematic Reviews and Meta-Analyses extension for Scoping Reviews) checklist, 22 articles were selected from 249 screened, focusing on medical context, objectives, data types, and visualization techniques. Published between 2003 and 2019, these articles primarily focused on clinical research, using various visualization methods such as timelines, temporal line charts, histograms, and scatter plots. These methods effectively simplified complexity through visualization and supported diverse medical objectives, delineating single patients, multiple patients, and cohorts. Cohorts were typically visualized in condensed form, whereas individual patient visualization tended to provide finer details. All systems enabled viewing and comparing patient data, although explicit comparison between single patients and cohorts was limited. Despite primarily using basic visualization techniques, some systems used new visualizations customized to specific tasks. Overall, the comparison of measurements between single patients and cohorts requires further systematic research and exploration in a design space.

O’Donoghue et al [13] focused on visualization of biomedical data. The visualized data spanned multiple biomedical areas, such as genomics, epigenetics, protein structures, cellular processes, and molecular interactions. This included 3D genomics, single-cell RNA sequencing, protein structure, phosphoproteomics, and metagenomics. Data were visualized through tailored visualizations integrating various datasets with supporting context to make the visualizations comprehensible to peers in the field. Techniques included the use of high–data density displays, color maps designed to reflect true data patterns, and the avoidance of poor practices such as rainbow color maps. Tools and methods such as parallel coordinate plots, heat maps, and 3D visualization techniques were used based on the type of data. Visualization methods were used to help address diagnostic errors, which often arise from improper cognitive processing of visual data, especially in visually intensive fields such as radiology. The aforementioned study suggests that better visualization practices could significantly reduce misdiagnoses, indicating the importance of these tools in clinical settings. While there are many powerful visualization tools available, they are underused in biomedical research. This underuse could be due to a lack of awareness or training on how to effectively use these advanced tools. The authors point out the need for improved education and resources so that these tools are more accessible and effectively used by more researchers and clinicians. It is suggested that visualization methods are currently not as widely used as they could be in hospitals, especially given their potential to reduce diagnostic errors.

### Conclusions

This scoping review revealed that, while numerous data visualization tools have been developed for tumor boards, many of them are either not adopted into routine practice or remain in the research phase. Omics data, particularly genomic information, are a major driver behind visualization innovation, yet the integration of these solutions into clinical workflows is often hindered by technical and organizational barriers. Tables remain a primary tool for visualizing clinical data, but there is potential for greater use of interactive and integrative visualization techniques. Moving forward, improving the accessibility and clinical integration of these tools, particularly through standardization and user-centered design, is crucial to ensuring their widespread adoption and enhancing multidisciplinary decision-making in oncology settings.

## Appendix

Multimedia Appendix 1 Search strategy: individual queries.

Multimedia Appendix 2 Publication distribution by year.

Multimedia Appendix 3 Distribution of the geographical origins of articles.

## Abbreviations

AMBAR: Alteration Annotations for Molecular Tumor Boards
MCC: multidisciplinary cancer conference
MTB: molecular tumor board
PRISMA: Preferred Reporting Items for Systematic Reviews and Meta-Analyses
PRISMA-ScR: Preferred Reporting Items for Systematic Reviews and Meta-Analyses extension for Scoping Reviews
