# Supplementary figures and images for: Data Visualization Support for Interdisciplinary Team Treatment Planning in Clinical Oncology: Scoping Review
Source: J Med Internet Res. 2025 Dec 9;27:e69104. doi: 10.2196/69104 (PMC12728401; doi:10.2196/69104)

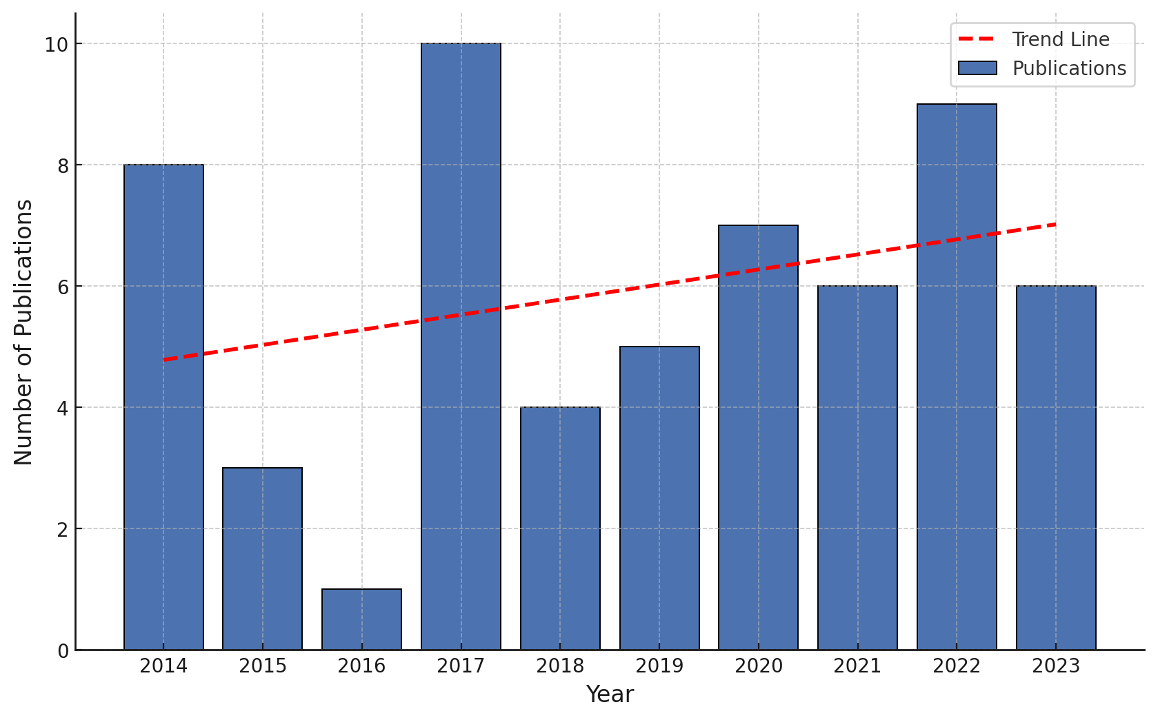

Supplement: Multimedia Appendix 2 [file jmir_v27i1e69104_app2.png]

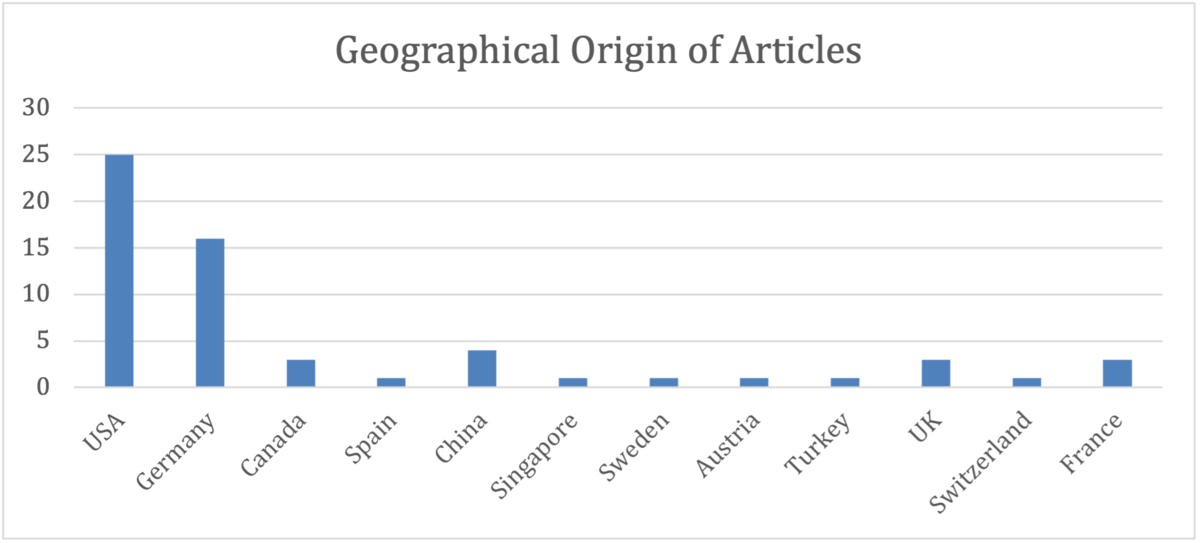

Supplement: Multimedia Appendix 3 [file jmir_v27i1e69104_app3.png]
